# Hyperbaric Oxygen Treatment Improves Intestinal Barrier Function After Spinal Cord Injury in Rats

**DOI:** 10.3389/fneur.2020.563281

**Published:** 2020-10-15

**Authors:** Xuehua Liu, Fang Liang, Jing Zhang, Zhuo Li, Jing Yang, Nan Kang

**Affiliations:** ^1^Department of Hyperbaric Oxygen Medicine, Beijing Chaoyang Hospital, Capital Medical University, Beijing, China; ^2^Department of Orthopedic Surgery, Beijing Chaoyang Hospital, Capital Medical University, Beijing, China

**Keywords:** hyperbaric oxygen, intestinal barrier, spinal cord injury, tight junction protein, Rho/ROCK signaling pathway

## Abstract

Intestinal barrier dysfunction is often observed clinically after spinal cord injury (SCI) and seriously affects long-term quality of life. Hyperbaric oxygen (HBO) treatment has been proved to promote barrier function recovery after injury, but the influence of HBO on intestinal barrier function following SCI is unclear. We aimed to investigate the effect and mechanisms of HBO treatment on intestinal barrier function by measuring the level of tight junction (TJ) proteins and the Ras homolog (Rho)/Rho-associated coiled-coil forming protein kinase (ROCK) signaling pathway. SCI model was established in rats, and the animals were randomly assigned into three groups: sham-operation group (SH), SCI group and SCI+HBO group. In the SCI+HBO group, the rats inhaled 100% O_2_ for 1 h at 2.0 atmospheres absolute pressure (ATA) once per day after surgery. Neurological function and intestinal permeability were assessed after surgery, and the jejunum tissue was excised for histological and intestinal barrier function evaluations. The protein levels of TJ and the Rho/ROCK signaling pathway were also measured. The results showed that in the SCI group, intestinal mucosal injury score, intestinal permeability, and levels of Rho and ROCK1 were higher, and TJ proteins occludin and ZO-1 were lower than those in the SH group (*P* < 0.01). HBO treatment significantly inhibited the expression of Rho and ROCK1, increased occludin and ZO-1 expression, decreased intestinal permeability, and alleviated intestinal mucosal injury as compared with the SCI group (*P* < 0.05, *P* < 0.01). The SCI+HBO group showed higher Basso–Beattie–Bresnahan (BBB) scores relative to the SCI group on postoperative days 7 and 14 (*P* < 0.01). There was a significant negative correlation between BBB score and intestinal mucosal injury score in rats after HBO treatment (*P* < 0.05). We concluded from this study that HBO treatment promoted the expression of TJ proteins possibly through inhibiting Rho/ROCK signaling pathway, which protected the intestinal barrier function and improved the intestinal permeability after SCI in rats.

## Introduction

Apart from the disastrous sensory and motor losses following spinal cord injury (SCI), dysfunction of a variety of organ systems is also widely recognized, such as the gastrointestinal (GI) tract, lung, kidney, spleen, and liver (1, 2). GI complications, e.g., barrier dysfunction, motility disorders, and microbial dysbiosis, reprehensively account for 11% of hospitalizations in SCI patients and are regarded as serious problems compromising quality of life (3, 4). Intestinal barrier dysfunction facilitates translocation of luminal bacteria into the circulation and mesenteric lymph, leading to life-threatening systemic inflammatory response syndrome (SIRS) and multiple organ dysfunction syndrome (MODS) (5). So far, some studies have reported intervention strategies to improve intestinal barrier function following SCI, such as melatonin, sacral nerve electrostimulation, and probiotics, which could promote intestinal peristalsis and suppress bacterial translocation (6–8). Hence, protection of intestinal barrier function is vital for intestinal homeostasis and the prognosis of SCI individuals.

Hyperbaric oxygen (HBO) refers to the inhalation of 100% pure oxygen in an environment >1 absolute atmospheric (ATM) pressure. Multiple studies reported that HBO treatment promoted barrier recovery after various injuries. HBO treatment could enhance the arterial partial pressure of oxygen (PaO_2_), increase the oxygen content, improve the perfusion of ischemia tissue, and stabilize the blood–brain barrier after cerebral ischemia/reperfusion injury (9, 10). Yang et al. demonstrated that HBO treatment stabilized the blood–spinal cord barrier by downregulating the expression of MMP-2 and MMP-9 following SCI (11). However, the influence of HBO treatment on intestinal barrier function following SCI is unclear, and the underlying mechanism needs to be clarified.

Tight junction (TJ) proteins, including claudin, occludin, and ZO-1, are the important components of intestinal barrier, which regulate the passage of ions and solutes, maintain cell polarity, and are the key determinants of the paracellular permeability (12–14). Ras homolog (Rho) belongs to G protein family, which circulates between an inactive guanosine diphosphate (GDP)-bound and an active guanosine triphosphate (GTP)-bound state and then regulates cellular activity through acting on its downstream target molecular, Rho-associated coiled-coil forming protein kinase (ROCK) (15). The Rho/ROCK signaling pathway has been proved to be an important regulator of TJ protein function (16, 17).

In present study, we aimed to investigate the effect and mechanisms of HBO treatment on intestinal barrier function after SCI in rats. We hypothesized that HBO treatment may promote the expression of TJ proteins through regulating the Rho/ROCK signaling pathway and may then protect intestinal barrier function.

## Materials and Methods

### Animals

All surgical operation and postoperative care were performed according to the ethical principles laid down by the Committee for Experimental Animal Welfare and Ethics, Capital Medical University. Healthy male Sprague–Dawley rats (*n* = 144, purchased from the Experimental Animals Center of Capital Medical University, Beijing, China) ≥8 weeks of age, weighing 250–300 g, were used in the experiment. The rats were feed with enough food and water in a stable temperature (23°C) and humidity (60%) with a 12:12-h light–dark cycle. Each rat was randomly assigned into three experimental groups: sham-operation group (SH), SCI group, or SCI+HBO group. Meanwhile, the experimental animals were also randomly assigned into four postoperative times (1, 3, 7, and 14 days). This study did not involve pathogenic microorganisms and was carried out in a general secondary laboratory, and all animals are specific pathogen free.

### Spinal Cord Injury Model

Contusive SCI was performed using a MASCIS (Multicenter Animal Spinal Cord Injury Study) impactor as described previously (18). The experimental rats were anesthetized using 10% chloral hydrate intraperitoneally (350 mg/kg) and underwent a laminectomy at level T10. The spinal cord dorsal was fully exposed and suffered a fall injury with a 10-g rod dropped from a height of 25 mm. After injury, the wound was sutured layer by layer, and the rats were placed in single cage and accepted intensive care to recover from anesthetic and surgical operations. We assisted the rats to empty their bladder twice a day until the spontaneous urination reflex was established.

### Hyperbaric Oxygen Treatment

Rats in the SCI+HBO group began to receive HBO treatment in an experiment hyperbaric chamber 6 h after operation. The chamber was flushed with 100% O_2_ for 5 min to avoid carbon dioxide accumulation. Compression air was conducted to 2.0 atmospheres absolute pressure (ATA) for 10 min and maintained for 60 min at 2.0 ATA inhaling 100% oxygen, then decompression air was performed to normobaric air for 5 min. According to the above treatment plan, rats received HBO treatment once a day. Rats in the SH and SCI groups inhaled 21% oxygen at 1.0 ATA postoperatively.

### Analysis of Locomotor Function

Locomotor function of rats was evaluated by the Basso–Beattie–Bresnahan (BBB) score for locomotion on postoperative days 1, 3, 7, and 14. The BBB scale includes the movement of hindlimbs and joints, weight-bearing capability, limb coordination and gait, paw position, and tail height. The highest score is 21. The behavior of rats was evaluated by two persons blinded to the experiment in an open field (120 × 120 cm) for 5 min.

### Assessment of Intestinal Permeability

On postoperative day 3, the intestinal permeability was evaluated by fluorescein isothiocyanate (FITC)-labeled dextran in the light of a method described previously (19). Briefly, rats (*n* = 12 per group) were prohibited from eating and drinking for 4 h and were given 0.6 mg/g of FITC-dextran (4 kDa; Sigma-Aldrich) dissolved in sterile phosphate-buffered saline (PBS) via gastric gavage prior to sacrifice. Four hours after gavage, the rats were anesthetized, and blood was collected via cardiac puncture. Specimens were centrifuged for 15 min at 3,000×*g*. The plasma was then measured using spectrofluorophotometer at an excitation wavelength of 490 nm and an emission wavelength of 525 nm.

### Tissue Harvest

At designated times after injury, experimental rats were deeply anesthetized using chloral hydrate. The abdominal cavity was exposed through a midline incision, and the small intestine was carefully separated. A 2-cm length of middle jejunum was obtained and fixed with 4% paraformaldehyde solution for histopathological assessment. Another part of the jejunum was placed in liquid nitrogen for protein analysis after flushing with saline.

### Histological Assessment

The jejunum was fixed in 4% paraformaldehyde overnight, flushed in PBS, and continuously dehydrated using low to high concentration of ethanol. After the ethanol in the tissue was replaced with xylene, tissue can be embedded in paraffin. After sectioning, 5-μm tissue sections were rehydrated again using orderly high to low concentration of ethanol; finally, the sections were put into the distilled water and stained with hematoxylin and eosin. Histological evaluation was performed by two pathologists blinded to this experiment protocol. The degree of intestinal mucosal injury was graded using a scale of 0–5 as described previously (20): 0 = intact intestinal villi and epithelium; 1 = subepithelial Gruenhagen's gap at the tip of villus, slight capillary dilation and congestion; 2 = moderate dilation of intestinal subepithelial space, separation of mucosa, and lamina propria; 3 = moderate to severe separation of mucosa and lamina propria, partial shedding of intestinal villi tips; 4 = shedding of intestinal villi and lamina propria, exposed dilated capillaries; and 5 = loss of intestinal villi, hemorrhage, and necrosis of lamina propria.

### Immunohistochemistry

The 5-μm tissue slices mentioned above were deparaffinized routinely; the slices were immersed in EDTA antigen repair buffer (pH 8.0) and heated in a microwave oven for antigen repair. After cooling naturally, the sections were washed with PBS (pH 7.4). In order to block the endogenous peroxidase activity, the sections were incubated in the dark for 25 min at room temperature in 3% hydrogen peroxide and then were washed with PBS (pH 7.4) again. The sections were incubated for overnight at 4°C with rabbit polyclonal anti-occludin (1:1,000, ab216327), rabbit polyclonal anti-ZO-1 (1:1,000, ab96587), rabbit monoclonal anti-Rho (1:1,000, ab40673), and rabbit monoclonal anti-ROCK1 (1:2,000, ab45171) (all from Abcam, Cambridge, MA, USA) as primary antibodies, then were added into horseradish peroxidase (HRP)-conjugated goat anti-rabbit IgG (Santa Cruz Biotechnology, Inc. USA) to cover tissue, and incubated for 50 min at room temperature. The sections were washed with PBS and added into DAB chromogenic liquid for color rendering, and the sections were counterstained with hematoxylin. The presence of brownish yellow granules was considered as positive protein expression. The average optical density (AOD) of positive staining in five fields *per section* and five sections per animal was analyzed using Image-Pro Plus 6.0 (Media Cybernetics, USA) software. All samples were analyzed by two double-blinded pathologists under high-times optical microscope.

### Western Blotting

In order to prepare the protein sample, the frozen jejunum tissue was lysed using tissue protein lysis buffer, and the protein concentration was determined according to the instructions of the bicinchoninic acid (BCA) protein quantitative kit (Sun Bio, Beijing, China). Sodium dodecyl sulfate–polyacrylamide gels (10–12%) were configured according to the weight of detected proteins. Fifty micrograms of protein in each sample was extracted to perform electrophoresis, transferred to nitrocellulose filter membranes, then was incubated overnight at 4°C with primary antibodies against occludin (1:1,000, ab216327), ZO-1 (1:1,000, ab96587), Rho (1:1,000, ab40673), ROCK1 (1:2,000, ab45171), and β-actin (1:2,000) (all from Abcam, Cambridge, MA, USA). After being washed three times, the membranes were incubated with HRP-conjugated secondary antibodies (Santa Cruz Biotechnology, Inc. USA) for 2 h at 37°C. Then the membranes were reacted using enhanced chemiluminescence (ECL) solution (Millipore, Corporation, Billerica, MA, USA), and then were scanned (Konica Minolta Medical Imaging, Inc., Wayne, NJ, USA). The expression of protein was quantitatively analyzed using target protein/β-actin with Adobe Photoshop (Adobe, Mountain View, CA, USA) and Lab Works (UVP, Upland, CA, USA) software.

### Statistical Analysis

The statistical analysis was performed by SPSS 19.0 (IBM Inc., Chicago, IL, USA) software. All data were expressed as the mean ± standard deviation (SD). One-way analysis of variance (ANOVA) was applied to analyze the differences among experimental groups in intestinal mucosal injury score, FITC level, locomotor score, and the expression of TJ and Rho/ROCK signal proteins. Pearson's correlation coefficient was calculated to analyze the relationship between locomotor function and intestinal mucosal injury. *P* < 0.05 was thought to be of significant difference in statistics.

## Results

### Hyperbaric Oxygen Treatment Maintains Intestinal Barrier Function After Spinal Cord Injury in Rats

**Figure 1:**
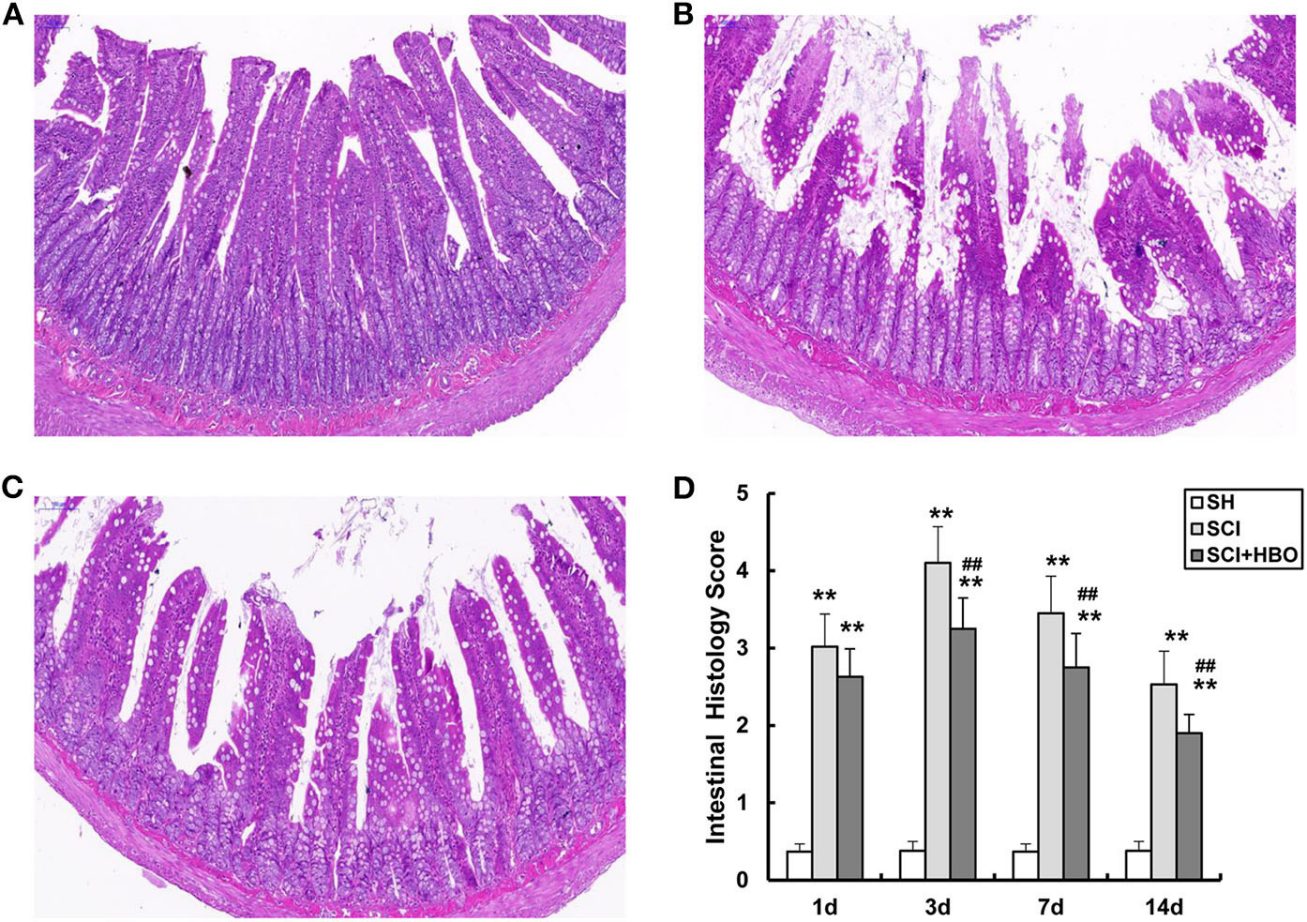
Representative photomicrographs of the jejunum stained with H&E on postoperative day 3 (all images are ×200 magnification, bar = 100 μm) and analysis of the jejunum mucosal tissue injury in experiment groups. **(A)** SH group jejunum showing normal villi and consistent villous height. **(B)** SCI group jejunum specimens were notable for marked short and disorganized intestinal villi, villi necrosis, and increased crypt depth. **(C)** SCI+HBO group animals showed no evidence of intestinal villi necrosis nor architectural deformity. **(D)** The histology score of jejunum. Data are presented as mean ± SD. Compared with that of the SH group, the histology score of jejunum was significantly higher in the SCI and SCI+HBO groups (**P < 0.01); HBO treatment significantly decreased the histology score compared with the SCI group (^##^*P* < 0.01). SH, sham operation; SCI, spinal cord injury; HBO, hyperbaric oxygen.

To evaluate the potential role of HBO treatment on intestinal mucosal histomorphology after SCI, the jejunum was removed from SCI rats at the appropriate times. As shown in Figure 1, compared with the SH group (Figure 1A), the jejunum mucosal tissue was compromised on day 3 following SCI (Figure 1B). For example, the villus height decreased significantly and the villi were disorganized, crypt depth increased significantly, the subepithelial interstice of villi was obvious, and upper cortex and solid layers were significantly detached. However, HBO treatment significantly decreased SCI-induced damage to the jejunal mucosa (Figure 1C). The mucosal injury score of randomly analyzed tissue segments was significantly elevated in the SCI group compared with the SH group (*P* < 0.01), and the rats in the SCI+HBO group exhibited a significantly lower mucosal injury score than that in the SCI group (*P* < 0.01, Figure 1D).

**Figure 2:**
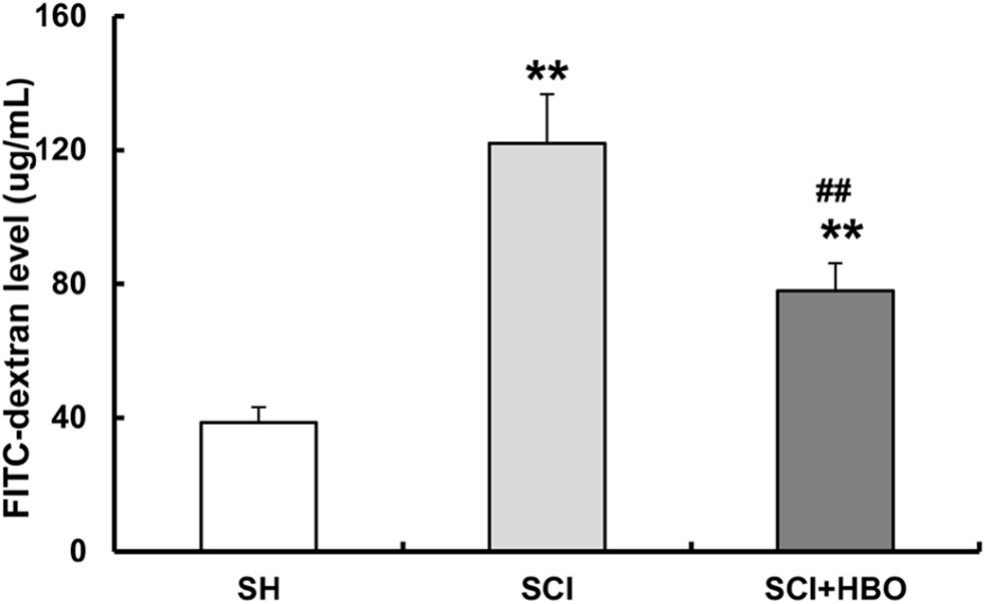
Effect of HBO on the intestinal permeability of SCI rats on postoperative day 3. Data are presented as mean ± SD. Compared with the SH group, there was a significant increase in the leakage of FITC-dextran from the gut in the SCI and SCI+HBO groups (**P < 0.01); HBO treatment significantly decreased the leakage of FITC-dextran compared with the SCI group (^##^*P* < 0.01). HBO, hyperbaric oxygen; SCI, spinal cord injury; FITC, fluorescein isothiocyanate.

To determine whether HBO treatment has any effects on intestinal barrier permeability after SCI, rats received FITC-labeled dextran (4 kDa) by gavage on day 3 following injury, and the FITC level in blood was measured. Intestinal permeability significantly increased after SCI. Remarkably, HBO treatment protected against injury-induced intestinal leakage (Figure 2). Taken together, these results demonstrated that HBO treatment improved intestinal barrier function.

### Hyperbaric Oxygen Treatment Increases the Expression of Occludin and ZO-1 After Spinal Cord Injury in Rats

**Figure 3:**
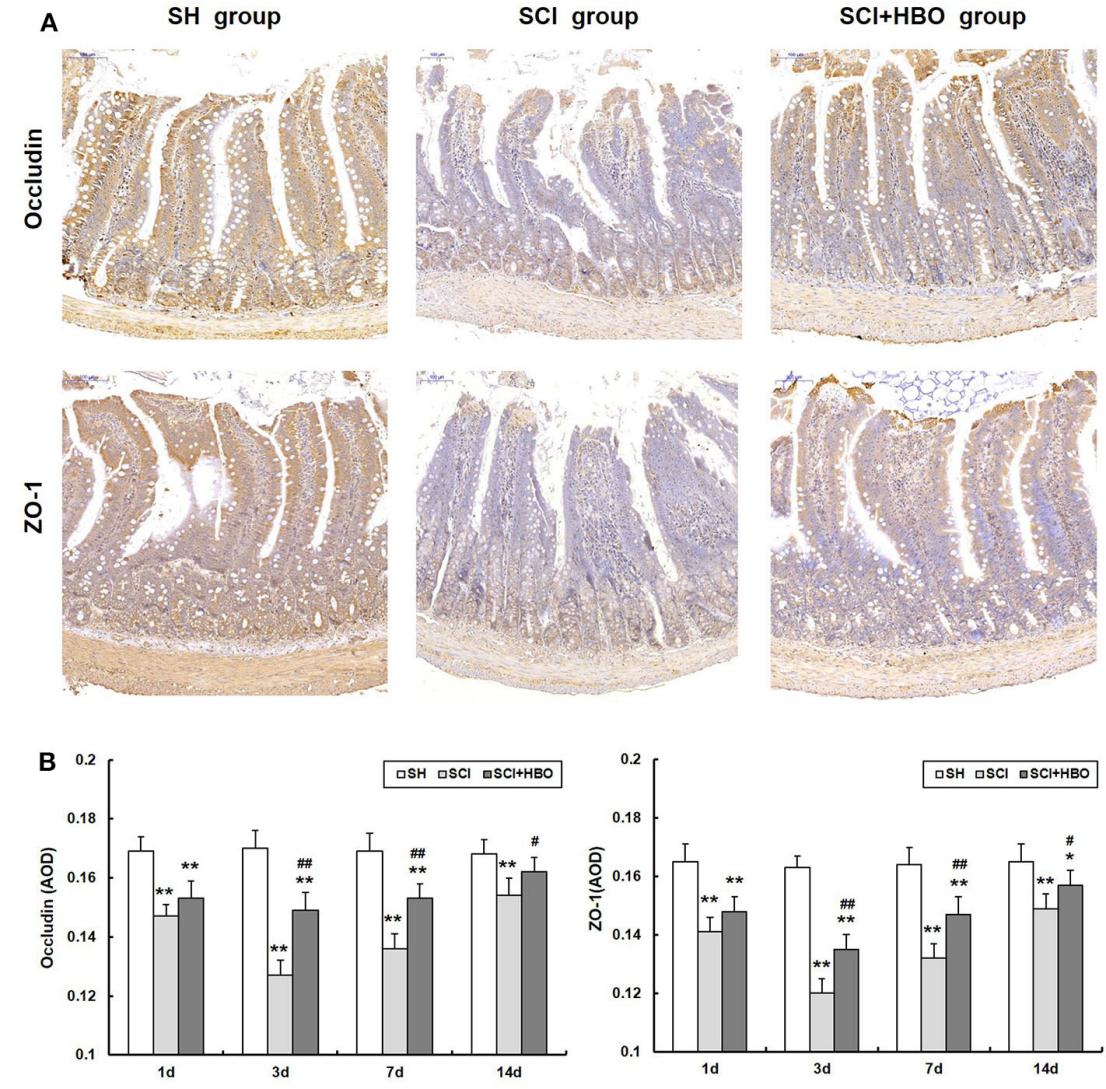
Effect of HBO on occludin and ZO-1 immunoreactivity in jejunum tissue after SCI. **(A)** Expressions of occludin and ZO-1 in the jejunum tissue visualized by immunohistochemistry under a high magnification on postoperative day 3 (all images are ×200 magnification, bar = 100 μm). **(B)** Analysis of the average optical density of occludin and ZO-1. Data are presented as mean ± SD. Compared with the SH group, the average optical density of two factors significantly decreased after SCI (*P < 0.05, **P < 0.01); HBO exposure significantly increased the average optical density of occludin and ZO-1 compared with the SCI group (^#^*P* < 0.05, ^##^*P* < 0.01). HBO, hyperbaric oxygen; SCI, spinal cord injury.

**Figure 4:**
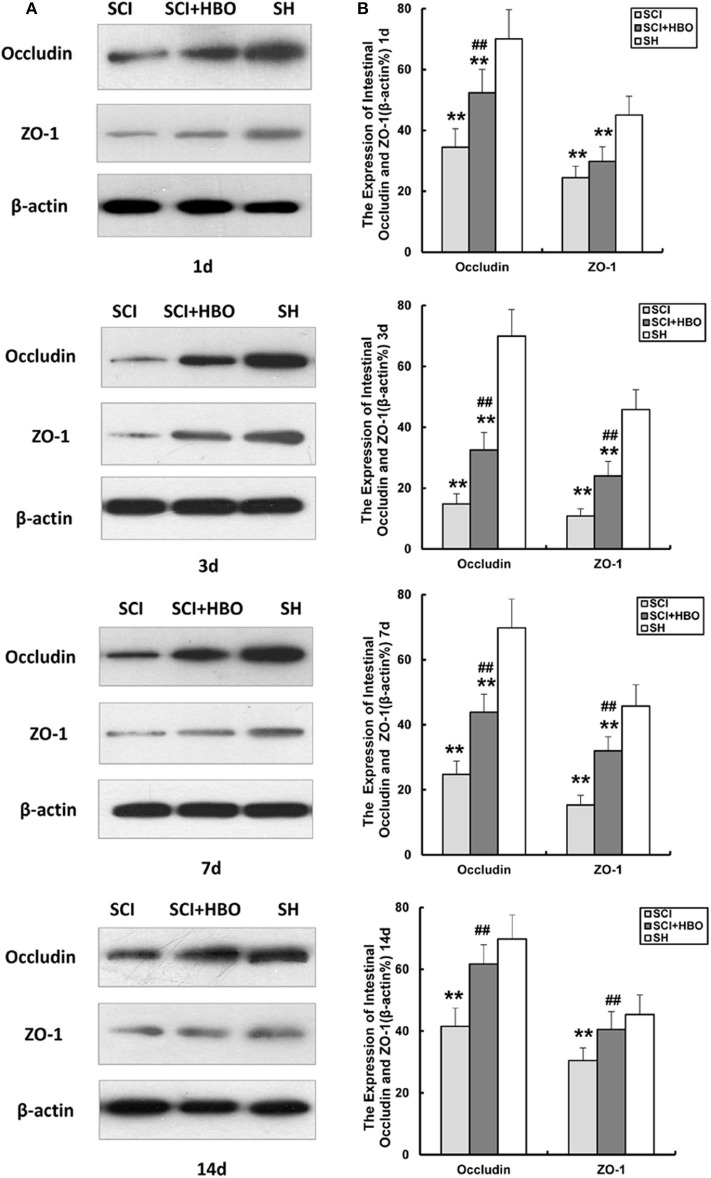
Effect of HBO on occludin and ZO-1 protein expression in jejunum tissue after SCI. **(A)** Representative immunoblot of two factors at different time points postoperatively. **(B)** Analysis of two factors of protein expression. Data are presented as mean ± SD. Compared with the SH group, the protein expression of two factors significantly decreased in the SCI and SCI+HBO groups (**P < 0.01); HBO treatment significantly increased their protein expressions compared with the SCI group (^##^*P* < 0.01). HBO, hyperbaric oxygen; SCI, spinal cord injury; SH, sham operation.

To assess the influence of HBO treatment on TJ proteins after SCI, we detected the expression of occludin and ZO-1 in jejunum tissue by immunohistochemistry and western blotting. For SCI rats, the expression levels of occludin and ZO-1 was significantly decreased compared with those of the SH group (*P* < 0.01), which decreased to a minimum level on postoperative day 3. However, for rats in the SCI+HBO group, the expression of these two proteins was higher than that in the SCI group on days 3, 7, and 14 postoperatively (*P* < 0.05, *P* < 0.01). Figure 3 shows the positive staining of occludin and ZO-1 detected by immunohistochemical method in different experimental groups, and the analysis of AOD. Figure 4 shows the protein expression level of occludin and ZO-1 determined by western blotting. The results suggested that HBO treatment upregulated the expression of TJ proteins after SCI.

### Hyperbaric Oxygen Treatment Downregulates the Expression of Rho and ROCK1 After Spinal Cord Injury in Rats

**Figure 5:**
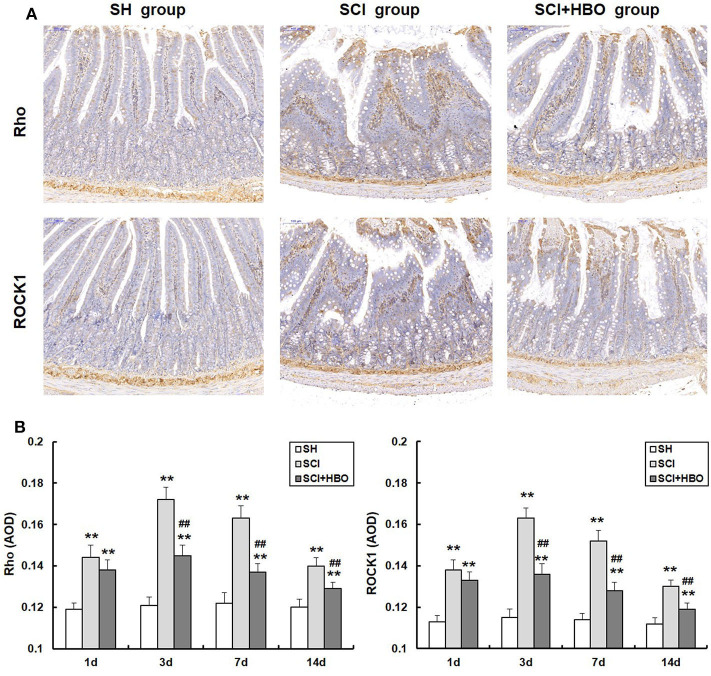
Effect of HBO on Rho and ROCK1 immunoreactivity in jejunum tissue after SCI. **(A)** Expressions of Rho and ROCK1 in the jejunum tissue visualized by immunohistochemistry under a high magnification on postoperative day 3 (all images are ×200 magnification, bar = 100 μm). **(B)** Analysis of the average optical density of Rho and ROCK1. Data are presented as mean ± SD. Compared with the SH group, the average optical density of two factors significantly increased after SCI (**P < 0.01); HBO exposure significantly decreased the average optical density of Rho and ROCK1 compared with the SCI group (^##^*P* < 0.01). HBO, hyperbaric oxygen; SCI, spinal cord injury.

**Figure 6:**
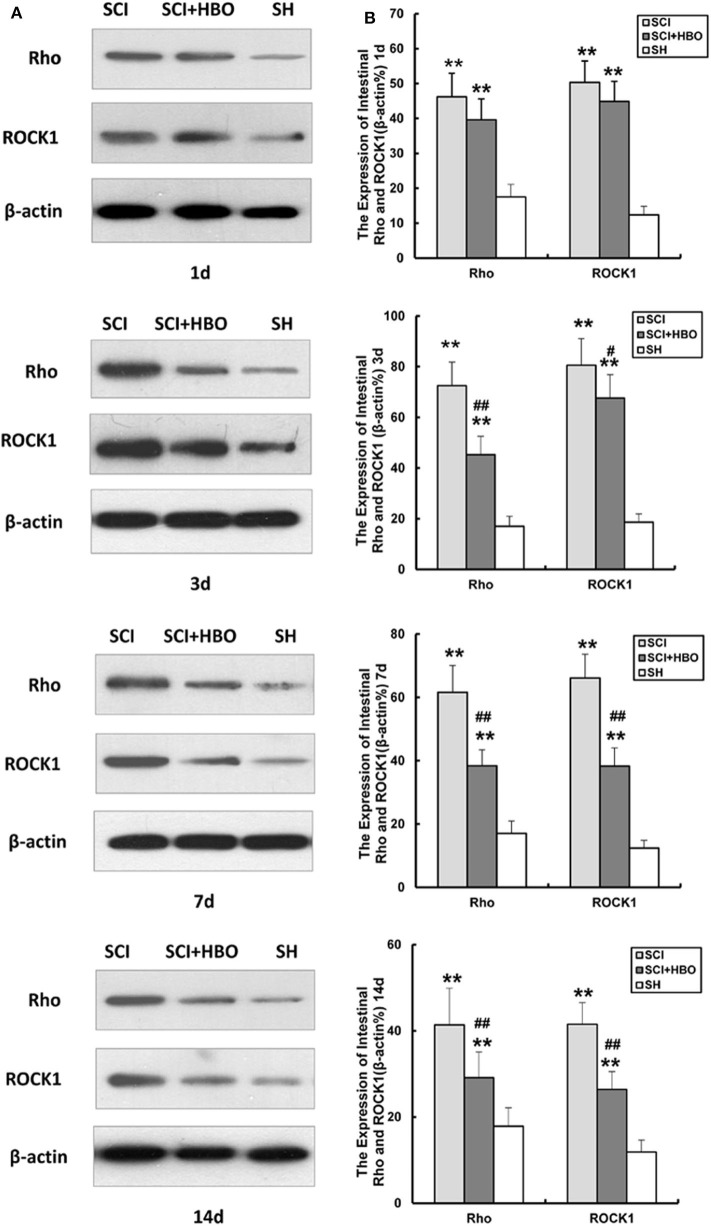
Effect of HBO on Rho and ROCK1 protein expression in jejunum tissue after SCI. **(A)** Representative immunoblot of two factors at different time points postoperatively. **(B)** Analysis of two factors of protein expression. Data are presented as mean ± SD. Compared with the SH group, the protein expression of two factors significantly increased in the SCI and SCI+HBO groups (**P < 0.01); HBO treatment significantly decreased their protein expressions compared with the SCI group (^#^*P* < 0.05, ^##^*P* < 0.01). HBO, hyperbaric oxygen; SCI, spinal cord injury; SH, sham operation.

In order to investigate whether HBO treatment influences the Rho/ROCK signal pathway, we analyzed the expression of Rho and ROCK1 in different experimental groups. As shown in Figures 5, 6, in the SCI group, the expression of Rho and ROCK1 was higher than that in the SH group (*P* < 0.01) and peaked on day 3 postoperatively. In contrast, the expression of Rho and ROCK1 was attenuated on days 3, 7, and 14 in the SCI+HBO group (*P* < 0.05, *P* < 0.01). These results suggested that HBO treatment inhibited the expression of Rho and ROCK1 after SCI.

### Hyperbaric Oxygen Treatment Improves Locomotor Recovery After Spinal Cord Injury in Rats

**Figure 7:**
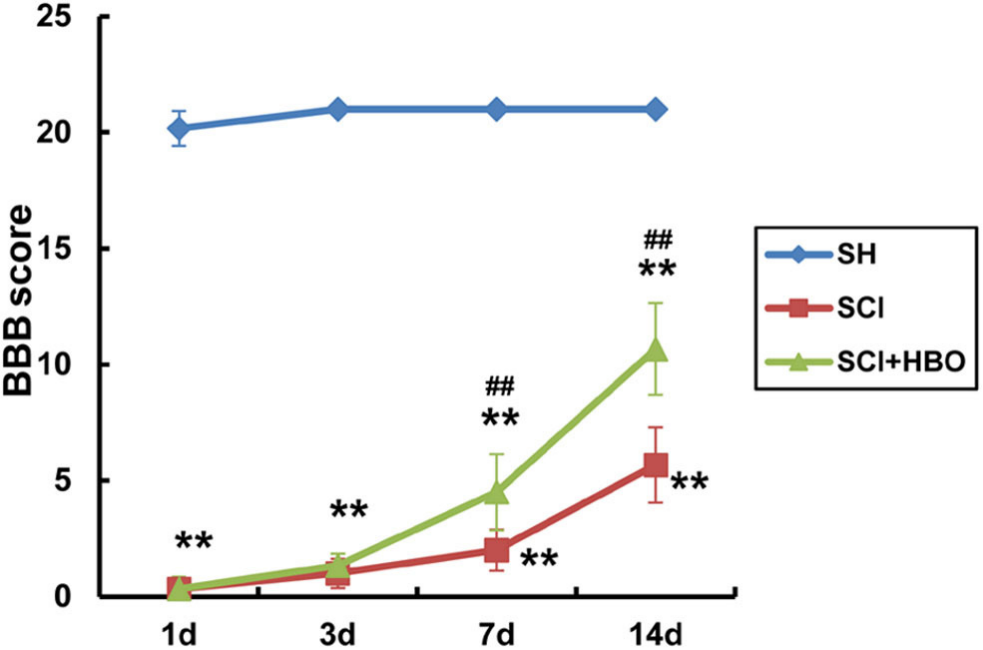
Evaluation of locomotor function of rats using BBB score in experimental groups at destined time points. Compared with that of the SH group, the BBB score of rats significantly decreased in the SCI and SCI+HBO groups at all destined time points (**P < 0.01); HBO treatment significantly increased the BBB score compared with the SCI group on days 7 and 14 postoperatively (^*##*^*P* < 0.01). BBB, Basso–Beattie–Bresnahan; SH, sham operation; SCI, spinal cord injury; HBO, hyperbaric oxygen.

Limb locomotor of rats was assessed using BBB rating scale to examine the effect of HBO treatment on locomotor recovery after SCI. Compared with the SH group, limb locomotor score of rats significantly decreased at the beginning and gradually increased over time in the SCI and SCI+HBO groups (*P* < 0.01). Rats treated with HBO exhibited a more rapid and greater improvement of locomotion than SCI rats. On days 7 and 14 postoperatively, the score in the SCI+HBO group was significantly higher than that in the SCI group (*P* < 0.01, Figure 7). These results indicated that HBO treatment promoted the recovery of motor function after SCI in rats.

### Correlations Between Locomotor Recovery and Intestinal Mucosal Injury

**Figure 8:**
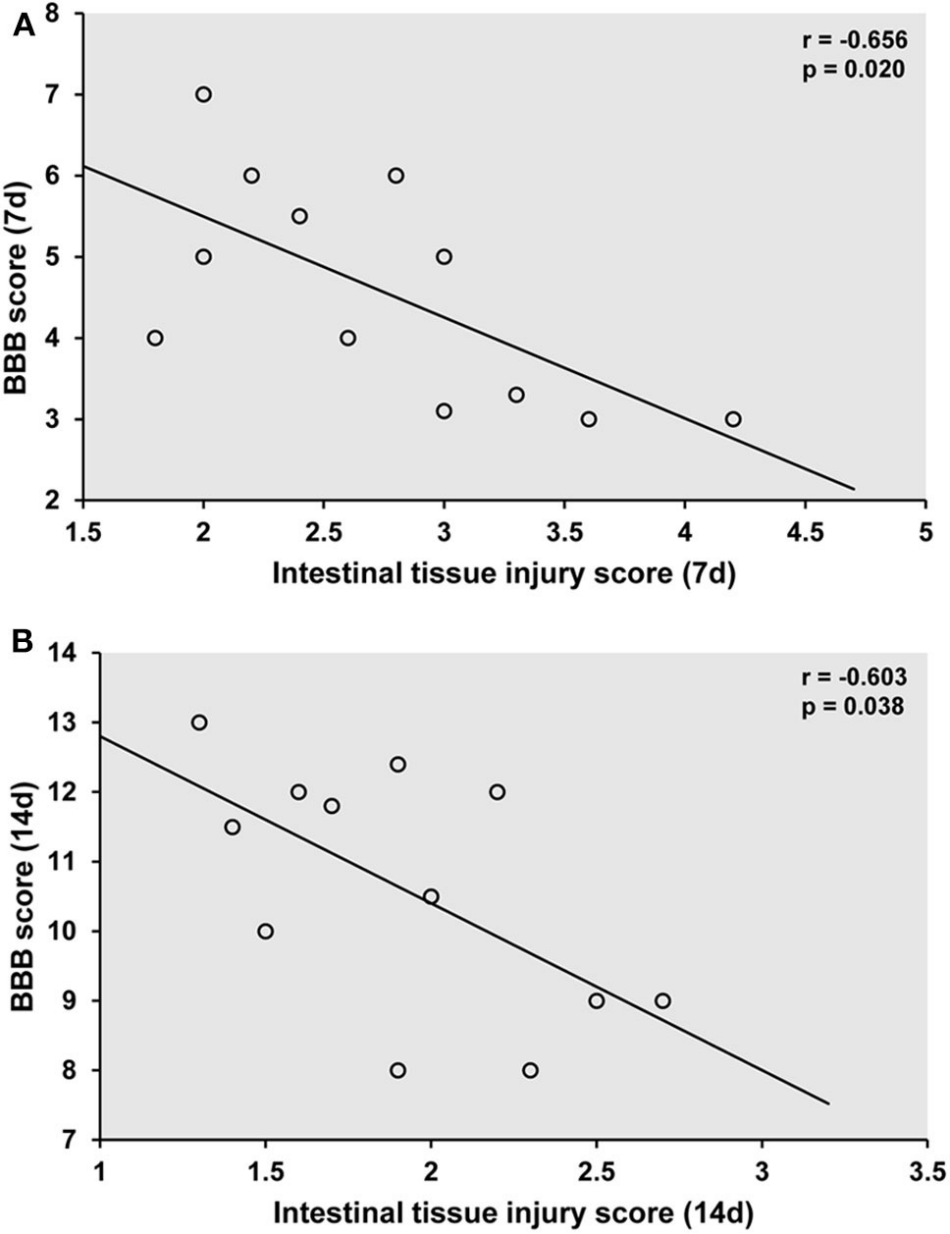
Association of locomotor recovery with intestinal mucosal tissue injury on postoperative days 7 **(A)** and 14 **(B)** in the SCI+HBO group. Pearson analysis showing the significant negative correlation between the recovery of locomotor function after HBO treatment and the degree of intestinal mucosal tissue injury in the SCI+HBO group (*P* < 0.05). SCI, spinal cord injury; HBO, hyperbaric oxygen.

We assessed whether locomotor recovery was linked to intestinal mucosal tissue injury by Pearson's correlation analysis. There was a significant negative correlation between BBB score and intestinal mucosal injury score in rats in the SCI+HBO group on days 7 and 14 postoperatively (*P* < 0.05); thus, greater locomotor recovery was significantly associated with less damage to intestinal mucosal tissue after HBO treatment (Figure 8).

## Discussion

Intestinal barrier dysfunction is a common complication of SCI, which severely reduces the quality of life of individuals with SCI (8, 21). Although few studies have shown that HBO treatment could protect intestinal barrier function in several animal models (22–24), it has not yet been investigated whether HBO treatment influences intestinal barrier function of SCI rats. In the present study, we examined the effect of HBO treatment on intestinal barrier function in a rat model of SCI. HBO treatment alleviated intestinal mucosal injury and protected intestinal barrier function. HBO treatment promoted the expression of TJ proteins to maintain the integrity of the intestinal mucosal barrier possibly by inhibiting the Rho/ROCK signaling pathway. In addition, we found that locomotor recovery of SCI rats was correlated with the degree of intestinal mucosal injury.

TJ is an important component and structural basis of intestinal mechanical barrier, which determines the intercellular permeability (25). A number of studies have shown that intestinal mechanical barrier could be strengthened by regulating TJ proteins. Park et al. demonstrated that theaflavins could increase intestinal barrier of Caco-2 Cell monolayers through increasing the expression of AMP-activated protein kinase-mediated occludin and ZO-1 (26). Peng et al. found that qihuang decoction could improve intestinal permeability through promoting the expression of TJ proteins after gastrectomy in rats (27), while following trauma and hemorrhagic shock, the metabolic changes of mucosal layer disrupted TJ protein expression, which could damage intestinal barrier and increase intestinal permeability (13).

The redistribution of systemic blood following SCI resulted in intestinal ischemia and hypoxia, which also disturbed the expression of TJ proteins and increased intestinal permeability, leading to intestinal barrier damage. After that, the bacteria and endotoxins shifted from intestine into blood, which caused enterogenous systemic infection and sepsis, even SIRS and MODS, and seriously influenced the patient's condition (21, 28). Consistent with previous studies, in the present study, intestinal mucosal injury score of rats was significantly increased after SCI. In addition, we found that the expression of occludin and ZO-1 was decreased, in parallel with an increase in intestinal permeability, which demonstrated that intestinal barrier was damaged after SCI.

As a noninvasive form of physical therapy, HBO treatment could protect intestinal barrier function though multiple mechanisms in several animal models. In a rat model of ischemia/reperfusion injury, HBO treatment improved intestinal barrier function by raising the partial pressure of oxygen and oxygen supply in blood, maintaining ATP and aerobic metabolism, and inhibiting TNF-α expression, therefore preventing intestinal epithelium from apoptosis (24). HBO treatment could activate genes encoding TJ proteins to maintain the integrity of intestinal barrier in colitis mice (22). In the sepsis rat model, HBO treatment alleviated intestinal barrier dysfunction of rats by altering the activation of NF-κB, nitric oxide, and myeloperoxidase (23). However, in the SCI rat model, it is unclear whether HBO treatment influences intestinal barrier function. In the current study, we found that HBO treatment significantly ameliorated intestinal mucosal injury of SCI rats. Improved intestinal barrier integrity was demonstrated by reduced gut leakage and restored expression of occludin and ZO-1 in HBO-treated rats. The underlying mechanism might involve the improvement of intestinal hypoperfusion and hypoxia after HBO treatment, which inhibited the decomposition and redistribution of TJ proteins (29). Detailed mechanisms of HBO treatment regulating the expression of occludin and ZO-1 remain to be clarified.

**Figure 9:**
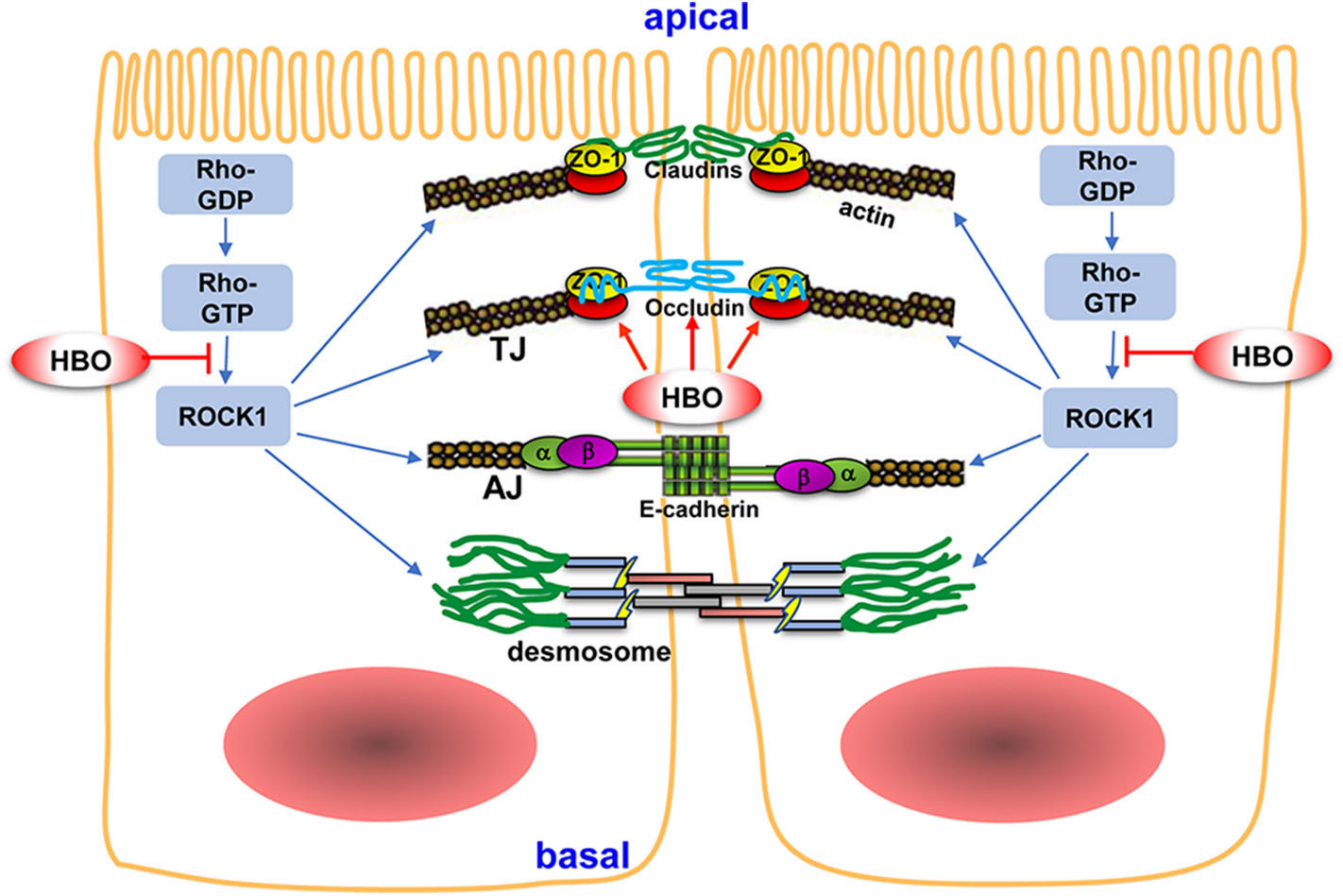
The potential mechanism of HBO treatment on intestinal barrier after SCI. The intestinal mechanical barrier mainly consisted of intact intestinal epithelial cells and their close connections. The connections between adjacent epithelial cells include tight junctions (TJs), adherent junctions (AJs), and desmosomes. TJs are mainly composed of claudins, occludin, and ZO. TJs are regulated by Rho/ROCK signaling pathway. HBO treatment increased the expression of occludin and ZO-1 following SCI and improved intestinal barrier integrity. Furthermore, HBO treatment significantly inhibited Rho/ROCK signaling pathway, which may be the mechanism of HBO treatment on regulating TJ proteins. Arrows indicate activation and lines with bars inhibition. For details, see text. HBO, hyperbaric oxygen; SCI, spinal cord injury.

Precise regulation of TJ proteins is essential to maintain intestinal barrier function. The Rho/ROCK signaling pathway plays an important role in the regulation of TJ proteins. Peng et al. found that qihuang decoction increased the expression of TJ proteins through inhibiting the Rho/ROCK signaling pathway after gastrectomy (27). In another study, Kim et al. demonstrated that the Rho/ROCK signaling pathway mediated the effect of hyaluronan on the expression of ZO-1 in mice (30). To investigate whether the Rho/ROCK signaling pathway was involved in HBO modulation on TJ proteins after SCI, we analyzed the expression of Rho and ROCK1 in different experimental groups. Compared with the SH group, SCI induced upregulation of Rho and ROCK1 expression in the intestinal tissue and downregulated the expression of occludin and ZO-1 (*P* < 0.01). Moreover, HBO treatment inhibited the expression of Rho and ROCK1 and increased the expression of occludin and ZO-1 accordingly (*P* < 0.05, *P* < 0.01). These results indicated that HBO treatment increases the expression of TJ proteins maybe through inhibiting the Rho/ROCK signaling pathway, which helps to improve intestinal barrier dysfunction induced by SCI in rats (Figure 9). The possible mechanism of HBO induced downregulation of the Rho/ROCK pathway might be that increased oxygen content inhibited the activation of Rho and ROCK in intestinal tissue after HBO treatment. In addition, oxidative stress was probably involved in this process (31, 32).

A lot of studies have indicated that translocated bacteria and endotoxins through damaged intestinal barrier induced systematic inflammation, which mediates the secondary injury after SCI and influences the recovery of neurological function (33–35). Meanwhile, these studies also hinted that protecting intestinal barrier might alleviate the degree of secondary injury after SCI. Previous studies have demonstrated that HBO treatment alleviated the inflammation and improved the recovery of neurological function after SCI (36–38). In present study, we also verified that HBO treatment improved locomotor recovery after SCI in rats. Furthermore, locomotor recovery was negatively correlated with intestinal mucosal injury after HBO treatment. These results further hinted that protecting intestinal barrier function and reducing intestinal mucosal injury maybe help in the recovery of neurological function after SCI.

## Conclusions

HBO treatment significantly improved neurological function, alleviated intestinal mucosal injury, maintained intestinal barrier integrity, and increased the expression of occludin and ZO-1 following SCI. Furthermore, HBO treatment significantly inhibited the Rho/ROCK signaling pathway, which might be the underlying mechanism involved in the regulation of TJ proteins by HBO treatment. In addition, locomotor recovery was negatively correlated with intestinal mucosal damage after HBO treatment. As far as we know, this study, for the first time, explored the effect of HBO treatment on intestinal barrier following SCI and the underlying mechanisms. These findings not only help to understand the molecular mechanisms of HBO treatment on improving intestinal barrier function but also provide the theoretical foundation for the clinical application of HBO treatment in GI complications following SCI. However, our study does have limitations. We only demonstrated that HBO treatment regulates the expression of TJ proteins and the Rho/ROCK signaling pathway after SCI, but the detailed mechanism of HBO treatment regulating their expression needs to be further investigated. Moreover, further studies are needed to fully translate these findings to human patients in order to expand the clinical application of HBO in SCI patients.

## Data Availability Statement

The raw data supporting the conclusions of this article will be made available by the authors, without undue reservation.

## Ethics Statement

The animal study was reviewed and approved by the Committee for experimental animal welfare and ethics, Capital Medical University.

## Author Contributions

XL, JY, and NK designed the experiments. XL, FL, and JZ performed the experiment and acquired the data. XL and ZL undertook the statistical analysis and interpretation of the data. XL wrote the first draft of the manuscript. JY helped to revise the manuscript. All authors read and have approved the final manuscript.

## Conflict of Interest

The authors declare that the research was conducted in the absence of any commercial or financial relationships that could be construed as a potential conflict of interest.
